# Protocols Used by Occupational Therapists on Shoulder Pain after Stroke: Systematic Review and Meta-Analysis

**DOI:** 10.1155/2021/8811721

**Published:** 2021-05-03

**Authors:** Isis Gabriele De Souza, Raphael Fabricio De Souza, Felipe Douglas Silva Barbosa, Kelly Regina Dias Da Silva Scipioni, Felipe J. Aidar, Aristela De Freitas Zanona

**Affiliations:** ^1^Occupational Therapy Department, Universidade Federal de Sergipe-UFS, Lagarto, Sergipe, Brazil; ^2^Physical Education Department, Universidade Federal de Sergipe-UFS, São Cristovão, Sergipe, Brazil; ^3^Occupational Therapy Department at Universidade Federal do Paraná-UFPR, Curitiba, Paraná, Brazil

## Abstract

**Introduction:**

Shoulder pain as a consequence after a stroke has multifactorial causes and can prevent the functional return of the upper limb. In addition, the effectiveness of clinical protocols applied by occupational therapists remains uncertain.

**Objective:**

To identify the main treatments currently used by occupational therapists for pain in the shoulder after a stroke.

**Method:**

Articles in English published between 2015 and 2019, of the randomized clinical trial type, with populations that stroke survivors a stroke and sequelae of shoulder pain were selected. The terms and combinations used were “shoulder pain and stroke and occupational therapy,” in the electronic databases, Directory of Open Access Journals (DOAJ), Occupational Therapy Systematic Evaluation of Evidence (OTseeker), and PubMed. Statistical Review Manager (version 5.3) established the significance level *P* ≤ 0.05.

**Results:**

Thirty-nine articles were found, but only four met the inclusion criteria. Electrical stimulation, therapeutic bandaging, and dry needling were eventually employed. For the meta-analysis, pain was the primary outcome, and range of motion (ROM) and upper limb function were secondary. Pain, ROM (external rotation, abduction, and flexion), and manual function were compared, and the meta-analysis showed improvement in the treatment group in clinical trials: pain (MD -2.08; 95% CI -3.23, -0.93; *P* = 0.0004), ROM (MD 4.67; 95% CI 1.54, 7.79; *P* = 0.0003), and manual function (MD 1.84; 95% CI 0.52, 3.16; *P* = 0.006).

**Conclusion:**

Dry needling, California tripull taping (CTPT), and functional electrical stimulation controlled by brain-machine interface (BCI-FES) are proved effective in shoulder pain and functionality.

## 1. Introduction

Pain is one of the disabling conditions that impairs involvement in significant occupations after a stroke. Specifically, pain in the shoulder-hand complex can prevent the functional return of the upper limb and generate stagnation in recovery. Shoulder pain after a stroke is present in most cases with sequelae, in about 50% to 80% of cases. It can be originated in the thalamus due to inefficiency of the system responsible for inhibiting pain or it can be of local origin (in the limb itself). Some people recover from shoulder pain after several months, but for others, it is a lasting problem [1].

Shoulder pain of local origin stems from multifactorial causes [2] and is associated with reduced motor function [3], decreased range of motion [4], and somatosensory impairments [5]. However, it is known that the main factors that cause shoulder pain after stroke result from sequelae secondary to injury, such as hemiparesis, which is mainly characterized by extreme loss of muscle strength and tenderness; unilateral negligence; injury to the rotator cuff tendons; reflex sympathetic dystrophy; subluxation of the shoulder; and spasticity or hypertonia [2, 6].

In addition to these factors, shoulder pain may have greater consequences than the discomfort caused by it, since the painful process interferes with functional capacity during activities of daily living [7–9].

Self-management interventions and gentle arm exercises were found to be effective [1]; however, the effectiveness of the protocols and which strategies are most used by occupational therapists in shoulder pain remains inconclusive. Thus, the aim of this research was to carry out a systematic review and meta-analysis, based on evidence, analyzing the main protocols currently used by occupational therapists to treat shoulder pain and facilitate independence in activities of daily living.

## 2. Methods

### 2.1. Analysis Methodology

The systematic review was carried out according to the PRISMA recommendation in the electronic databases Directory of Open Access Journals (DOAJ), Occupational Therapy Systematic Evaluation of Evidence (OTseeker), and PubMed. Databases were consulted between the months of August and October 2019. The terms and combinations, respectively, used for search are derived from the Health Sciences Descriptors/DeCS: shoulder pain, stroke, and occupational therapy.

### 2.2. Inclusion and Exclusion Criteria

It was selected articles in English published between 2015 and 2019, only randomized clinical trial studies, with populations that suffered a stroke and sequelae of shoulder pain. A study was excluded if it addressed treatments that were not applied by occupational therapists, it did not recommend a clinical practice of occupational therapy, it was a duplicate, and it used protocols applied to animals. The object of this study was to identify protocols used by occupational therapists to treat shoulder pain resulting from stroke.

### 2.3. Eligibility Criteria

Data collection was carried out independently; the material collected respected the following steps: title of the study refined by the subject, type of study (randomized clinical trial), and reading of the abstract. Articles that did not meet the eligibility criteria were excluded. After the first filter, a complete reading of the studies was performed.

The primary outcome used was shoulder pain, verified by the Visual Analog Scale (VAS) and algometry. Secondary outcomes were shoulder range of motion (external rotation, abduction, and flexion) and upper limb function.

### 2.4. Methodological Quality Assessment

All selected articles were evaluated using the Jadad scale [10], an instrument that quantifies the methodological quality of randomized clinical trials. This scale measures the adequacy of randomization, double-blinding, and losses of study participants. A score with a value equal to or greater than 3 is considered good quality.

### 2.5. Statistical Analysis

The results were presented as mean difference (MD) with 95% confidence intervals (CI), presented by the forest plot graph. For meta-analysis, it was used groups: control (exercises and functional activities commonly used in clinical practice) and experimental (intervention with equipment or resources associated with exercises and functional activities to treat shoulder pain).

Shoulder pain, range of motion, and upper limb function were assessed. For range of motion assessment, 3 subgroups (abduction, shoulder flexion, and external rotation) were used. For upper limb function, 2 subgroups (Fugl-Meyer assessment and manual function test) were used. Heterogeneity was quantified by Cochran's test (Ch^2^) that quantified the inconsistency (percentage of the total variation of studies by heterogeneity) of effects by *I*^2^ statistics. We use a random model for the analysis; since the levels of a factor from a population were captured at random, we assume that the individual effects are randomly distributed around an average [11]. The statistical program Review Manager (version 5.3) was used, with a significance level of *P* ≤ 0.05.

## 3. Results

**Figure 1:**
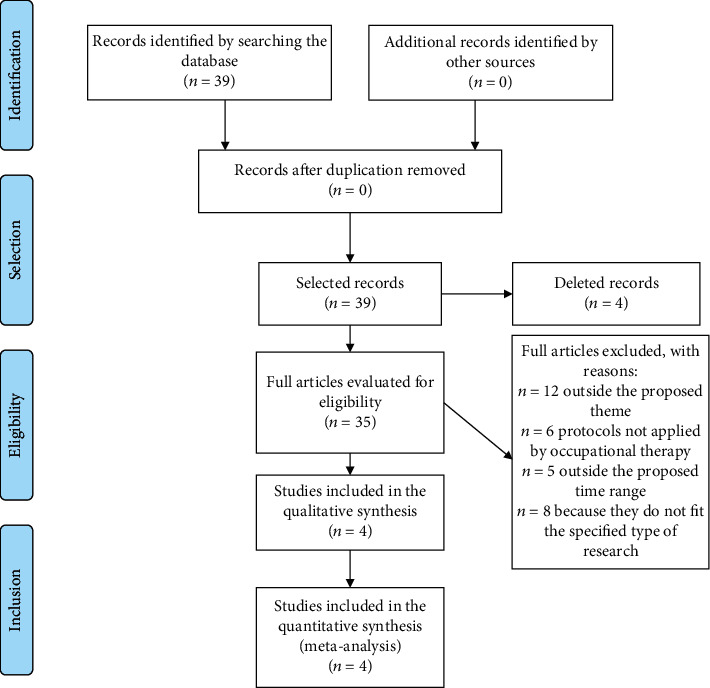
Research flowchart according to PRISMA recommendation.

A total of thirty-nine articles were found, of these thirty-two in the PubMed database, four in the DOAJ database, and three in the Otseeker database. After reading the title and abstract, four articles were excluded because they were outside the proposed theme. After reading the full text, 31 articles were excluded: twelve for being outside the proposed theme, six for being protocols not applied by occupational therapists, five for being outside the proposed time range, and eight for not being in the type of research specified. Only four articles were included in this study (Figure 1); the four studies were included because they are techniques that were applied by occupational therapists in the intervention protocol described.  Table 1 shows the matrix of results achieved.

Protocols for the treatment of painful shoulder using the techniques were identified in the literature: electrical stimulation-transcutaneous electrical nerve stimulation (TENS) and transcutaneous neuromuscular electrical stimulation (t-NMES) [12], functional electrical stimulation controlled by brain-computer interface (FES-BCI) [13], therapeutic taping called California tripull taping (CTPT) [14], and dry needling [15].

### 3.1. Meta-Analysis

For the meta-analysis, the individual and summarized effects of the studies that evaluated shoulder pain (*n* = 2), ROM (*n* = 3), and upper limb function (*n* = 2) were evaluated. The general analysis showed improvement in the treatment group in the outcomes: pain (MD -2.08; 95% CI -3.23,-0.93; *P* = 0.0004), ROM (MD 4.67; 95% CI 1.54, 7.79; *P* = 0.0003), and upper limb function (MD 1.84; 95% CI 0.52, 3.16; *P* = 0.006).

When these variables were evaluated by subgroups, the ROM showed improvement in shoulder flexion (MD 5.14; 95% CI 1.74, 8.75; *P* = 0.003), no differences in abduction (MD 1.81; 95% CI -9.34, 12.97; *P* = 0.75), and external rotation (MD 2.48; 95% CI -8.53, 13.48; *P* = 0.66); in the upper limb function, there was an increase in the Fugl-Meyer score (MD 1.80; 95% CI 0.46, 3.14; *P* = 0.009).

**Figure 2:**
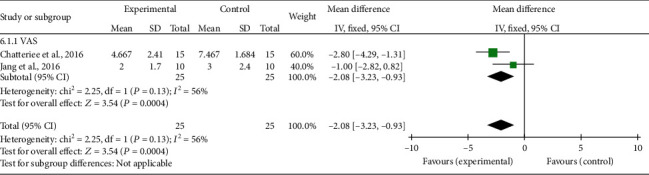
Shoulder pain: forest plot of the articles that used the Visual Analog Scale (VAS). Legend: SD: standard deviation; CI: confidence interval; VAS: Visual Analog Scale.

**Figure 3:**
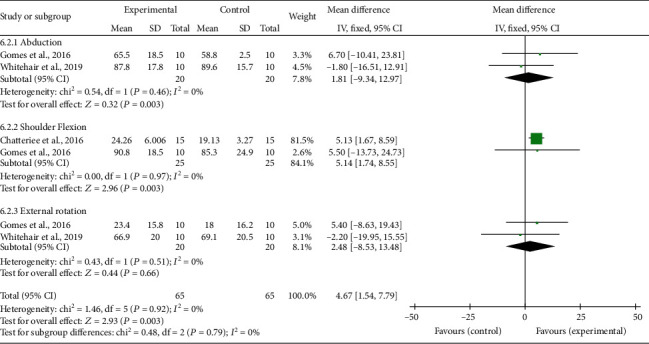
Range of motion: forest plot of the articles that used goniometry of the flexion, abduction, and external rotation movements of the shoulder. Legend: SD: standard deviation; CI: confidence interval.

**Figure 4:**
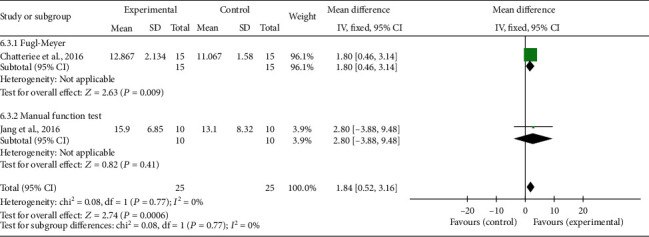
Upper limb function: forest plot of articles that used Fugl-Meyer and manual function test. Legend: SD: standard deviation; CI: confidence interval.

Evidence of heterogeneity and inconsistency was found for pain (Ch^2^ = 2.25; *I*^2^ = 56%). On the other hand, ROM and upper limb function showed a percentage of the total variation of studies due to null heterogeneity (*I*^2^ = 0%). All results can be seen in Figures 2–4.

The studies were analyzed for risk of bias by the funnel plot model; however, the included studies did not show publication bias.

## 4. Discussion

The results of this review support that electrical stimulation (with and without control by brain-machine interface), therapeutic taping, and dry needling are the main protocols used by occupational therapists in shoulder pain. The results of the meta-analysis indicated pain reduction. Upper limb function and ROM proved to be favored by these treatments. Taken together, these results suggest that poststroke people can restore their daily routines in short periods.

When analyzing the protocol periods, it was found that the average intervention time of the studies was 6 weeks for the electrical stimulation technique associated with BCI and also for the California tripull taping; in dry needling intervention, individuals received both interventions separated by at least 15 days. Each intervention was applied once a week for 3 weeks or more, and 10 sessions for the application of TENS and t-NMES. However, better understandings of the outcomes found can be verified according to the technical specificities of each protocol, presented below.

### 4.1. Electrical Stimulation

Electrical stimulation is applied by fixing electrodes at motor points on the skin surface. The conduction of the electric current is capable of producing pulses with variable frequencies that go beyond the surface of the skin and consequently influences the mechanism of pain conduction through nerve pathways. Depending on the modulation of the applied current, it activates different pain response mechanisms. When used under low frequency and high intensity, it aims to activate opioid nociceptors in order to promote the release of endogenous analgesic substances by the nervous system. On the other hand, when applied at high frequency and low intensity, the fast-acting A*β* afferents (sensory stimuli in mechanoreceptors) can inhibit the painful stimuli to pass through the slow-moving afferent A*δ* and C pathways [6].

In the studies that addressed this methodology, it was observed some technical peculiarities. In the research done by Whitehair et al. [12], the position of the electrodes was inserted every two inches into the contractile units of the middle deltoid and upper trapezius muscles of the affected shoulder with the frequency parameters of 100 Hz and pulse of 300 microseconds. For TENS, sensory nerves were stimulated without producing muscle contraction, with a frequency of 35 Hz, duration of 300 microseconds, with acceleration of 2 seconds and deceleration of 2 seconds, for t-NMES. The study that aimed to compare high and low currents showed negligible results for pain-free passive ROM between t-NMES and TENS currents. It is speculated that greater intervals in the application of electric currents may achieve such benefits [12].

Previous studies have identified short-term changes in motor-evoked potential and cortical blood flow as an effect of peripheral electrical stimulation [16–18]. The application of TENS to the median nerve, performed during MRI in healthy individuals, has been shown to activate the main sensory and motor regions of the brain in the contralateral hemisphere to stimulation [19]. In addition, pioneering studies have shown that the sustained maintenance of sensory input affected the representations of maps in the somatosensory cortex [20, 21] associated with the recovery of sensorimotor deficits and, consequently, changes in pain perception [22, 23].

Sensory restoration by electrical stimulation comprises the activation of axonal membranes available to trigger a train of action potentials in response to an exogenous electrical stimulus. Such a stimulus needs to be performed in a way that the brain interprets it as coming from a real endogenous sensory receptor, in other words, corresponding to response profiles of natural receptors. In general, stimulation with a square pulse (or a train of square pulses) has become the gold standard as it can provide rapid depolarization of the axonal membrane and it is relatively easy to implement [24].

A single session of peripheral electrical stimulation applied to a paretic handled to transient improvements in grip strength [25, 26] and facilitated the effects of cortical plasticity and motor function in patients with chronic and subacute stroke [27, 28].

However, Jang et al. [13] comparing BCI-controlled by functional electrical stimulation (FES) point out that limitations were identified when applied to electrical stimulation alone, for keeping participants passive during therapy, generating a deficiency in sensory and motor reeducation. Jang et al. [13] propose that the use of FES with BCI promotes a type of neurofeedback that stimulates the sensorimotor system, promoting the active participation of the subject in various activities [13].

The association of electrical stimulation controlled by BCI is a potential tool to decrease pain and also to improve upper limb function after stroke. The BCI equipment consists of EEG sensors—they capture information from brain waves connected with the FES device. The BCI technique through the EEG performs the measurement of neuronal signals, processes the amplification of this information, and classifies them in an algorithm. These data will feed the device capable of producing electric current (FES), which stimulates movement through pulses of energy promoting contraction of muscle fibers through biofeedback. The principle of this protocol is verified by the motor imagery (produced by the imagination of movements) and a precise neural signal, triggering the FES to perform isolated movements, producing neuroplasticity of brain structures affected by stroke [13].

In the aforementioned study, the application of the protocol occurred associated with the conventional occupational therapy treatment (not clarified by the authors) plus the FES and FES-BCI for both groups with a duration of twenty minutes. The FES parameters were duration 15 seconds with an interval of 7 seconds, frequency of 35 Hz, and pulse of 150 microseconds, with intensity modulated in line with the biofeedback produced in the joint. Below this threshold, FES activation did not occur. There was electrical stimulation associated with the concentration level. The results showed an improvement in shoulder subluxation, flexion, and abduction movements in the FES-BCI group compared to the FES group. However, both groups showed improvement in pain and functional mobility. This suggested that the analgesic mechanisms of electrical stimulation stimulated the opioid receptors or the fast-acting A*β* afferent pathway. Thus, electrical stimulation was shown to be intensified when used together with the treatment of cognitive aspects such as FES-BCI [13].

### 4.2. Taping

Taping uses self-adhesive tapes applied in specific regions. These tapes aim to produce sensitive cutaneous inputs, guided by sensory nerve pathways to the brain [29]. Depending on the positioning techniques applied towards the fibers or against, responses in the activation or inhibition of the muscles will be verified, in addition to supporting articular structures [30].

The taping has been applied in order to stabilize the joint, inhibit muscle activity, reduce pain, and increase joint torque and the excitability of motor neurons [30]. It is one of the techniques used that significantly involves the sensory system due to its relation of importance in the afferences to the structures that compose and manage motor control, movement performance, and pain. For a better motor response, it must have more effective sensory inputs. Therefore, the sensory system is essential to capture, conduct, and perceive the performance of movements [31].

In the study by [14], two types of tapes were used: 1.5 cm adhesive cotton and 1 cm rigid cotton. In this case, it is aimed at bringing the humerus head closer to the glenoid fossa, in order to reduce pain by preserving the structure. The application was made by three cotton shavings positioned under the skin without exerting traction, for the protection of the cutaneous surface, then three rigid shavings applied under the tapes to exert traction, California Tri-pull taping (CTPT) method. Two sets of tapes were placed at the ends of the chips for better adherence. Both groups evaluated received conventional neurorehabilitation treatments, lasting 45 minutes, five days a week for six weeks, but the experimental group received additional treatment with taping, three times a week during the same period. The results showed that subluxation and pain were attenuated in addition to showing improvement in upper limb flexion and function.

In this study, it is believed that the pain reduction occurred due to the protection of the articular structure through the tape, against additional injuries, during the movements of ambulation and performing activities of daily living. It was done by the rapprochement of the head of the humerus to the glenoid fossa, in addition to improvement of sensory inputs for the region resulting in improved muscle function, as well as increased local vascularization. It was concluded that the CTPT method is efficient in reducing shoulder pain, improving flexion and distal functioning of the upper limb.

### 4.3. Dry Needling

The study done by Mendigutia-Gomez et al. [15] is aimed at proving the effectiveness of dry needling in reducing upper limb spasticity resulting from stroke and, consequently, reducing pain. The treatment period occurred for 3 weeks, 1 session per week. All participants received conventional rehabilitation to treat spasticity (not specified by the authors). Arm exercises were used to decrease muscle tone in task training and positioning of the shoulder girdle for 45 minutes. Although the results did not reveal improvements in the spasticity of trapezius, pectoralis major, and subscapularis muscles, it was reduced in the infraspinatus muscle, decreasing the sensitivity of the pain and increasing the amplitude of movement of abduction and external rotation.

On the other hand, in a previous study, it was demonstrated that the application of dry needling caused post-needling-induced pain in 50% of patients who suffered a stroke, but it was absent after 72 h of intervention without any additional therapeutic action. In addition, the inclusion of dry needling in a rehabilitation session was effective in reducing the intensity of shoulder pain in this population [32]. Previous studies have found that the application of acupuncture is effective in reducing symptoms of shoulder pain among people who have suffered from stroke [33].

The mechanisms underlying the changes observed in shoulder pain after applying dry needling are not clear, but some hypotheses are proposed [34]. Dry needling is supposed to have several mechanical triggering effects on the central nervous system, and a cascade of neurophysiological signaling leading to antinociceptive effects modulating the activity of the spinal dorsal horn and activating the central pain inhibitory pathways [35]. These effects could explain the changes observed in the intensity of shoulder pain.

### 4.4. Meta-Analysis

The meta-analysis showed that the protocols of CTPT and electrical stimulation combined with the brain-machine interface (BCI) were effective in reducing pain. The protocols that used the techniques of CTPT and dry needling showed an increase in ROM specifically for shoulder flexion, since it is an important movement performed in basic daily activities and performed with restriction when there is pain in amplitude greater than 90°. For the outcome of the upper limb function in the protocols of CTPT and functional electrical stimulation (FES) controlled by BCI, both techniques were efficient to increase motor function, especially in everyday simulation movements.

We identified as an important limitation of our study, the short search period for the studies. In fact, the search made between 2015 and 2019 may restrict other findings from previous years; however, as the objective of this work was to identify the main protocols of today, we suggest that future research can make a historical retrospective of which strategies and tools were used by occupational therapists to treat shoulder pain.

Only at most four randomized clinical trials (RCT) were pooled to generate the meta-analysis; this can be considered as another important limitation for the current meta-analytic study. We suggest future directions of conducting more eligible RCT for future meta-analysis on the same research topic.

## 5. Conclusion

Recurrent shoulder-hand complex pain in people who have suffered a stroke has multifactorial causes, often associated with hemiplegia, subluxation, or spasticity, conditions that reduce the functionality of the upper limb. The pain process directly interferes with participation in daily life, in the routine of activities, in rest, in sleep, and in mood. Occupational therapists facing this problem may have protocols for treating this condition.

All protocols studied showed improvements in shoulder pain after stroke, besides the ROM and upper limb function. They have been pointed out as recurrent methods of occupational therapists: electrical stimulation, taping through the California tripull taping, electrical stimulation associated with the brain-machine interface, and dry needling.

However, we know that occupational therapists use several other protocols in their clinical practice, but the visibility of actions may be impaired by the lack of use of occupational therapy terms in titles and keywords in national and international publications. Thus, it is also encouraged that occupational therapists adopt an evidence-based practice in their clinical work, but also taking into account the specificities of each subject.

## Figures and Tables

**Table 1:** Protocols used by occupational therapists to treat a painful shoulder.

Author	Research title	Year	*N*	Protocol	Details of the intervention	Results	Score from Jadad
Whitehair et al.	The Effect of Electrical Stimulation on Impairment of the Painful Post-Stroke Shoulder	2019	10	Transcutaneous electrical nerve stimulation (TENS) Transcutaneous neuromuscular electrical stimulation (t-NMES)	Subjects were treated randomly with TENS and t-NMES. Passive external rotation without pain and the abduction range of motion of the affected shoulder during stimulation were measured.	There were no significant differences between subjects in range of motion without pain for external rotation or abduction. This demonstrates the lack of an acute effect of TENS and t-NMES in reducing pain.	3
Mendigutia-Gomez et al.	Effect of Dry Needling on Spasticity, Shoulder Range of Motion, and Pressure Pain Sensitivity in Patients With Stroke	2016	20	Dry needling (DDN)	Control and experimental group. The experimental group received the standard treatment combined with DDN on the subscapular infra-spinal trapezius muscles and the pectoralis major muscle on the spastic shoulder.	Patients who received DDN exhibited improved range of motion due to decreased spasticity in the infraspinatus muscle. There was a significantly higher increase in all pressure pain thresholds, abduction and external rotation of the shoulder, after DDN.	5
Chatterjee et al.	The California Tri-pull Taping Method in the Treatment of Shoulder Subluxation After Stroke: A Randomized Clinical Trial	2016	30	California tripull taping (CTPT)	All participants received conventional neurorehabilitation 5 days a week for 6 weeks, half of the participants also received CTPT.	The CTPT method demonstrated a significant reduction in pain in the treatment group, significant improvement in active shoulder flexion, and significant improvement in proximal arm function.	2
Jang et al.	Effects of Brain-Computer Interface-Controlled Functional Electrical Stimulation Training on Shoulder Subluxation for Patients with Stroke: A Randomized Controlled Trial	2016	20	Functional electrical stimulation (FES) controlled by brain-computerinterface (BCI)	20 subjects were randomly divided into two groups: the BCI-FES group (*n* = 10) and the FES group (*n* = 10). Patients in the BCI-FES group received conventional therapy with BCI-FES in the subluxation area of the shoulder of the paretic upper extremity, five times a week for 6 weeks, while the FES group received conventional therapy with the FES.	The BCI-FES group demonstrated significant improvements in the assessments of pain and manual function used. The results of this study suggest that training with BCI-FES can be effective to improve the subluxation of the shoulder of stroke patients, facilitating motor recovery.	3

Legend: stroke: TENS: transcutaneous electrical nerve stimulation; t-NMES: transcutaneous neuromuscular electrical stimulation; DDN: dry needling; CTPT: California tripull taping; FES: functional electrical stimulation; BCI: brain-computer interface.

## References

[1] Lindgren I., Gard G., Brogardh C. (2018). Shoulder pain after stroke - experiences, consequences in daily life and effects of interventions: a qualitative study. *Disability and Rehabilitation*.

[2] Wilson R. D., Chae J. (2015). Hemiplegic shoulder pain. *Physical Medicine and Rehabilitation Clinics of North America*.

[3] Adey-Wakeling Z., Arima H., Crotty M. (2015). Incidence and associations of hemiplegic shoulder pain poststroke: prospective population-based study. *Archives of Physical Medicine and Rehabilitation*.

[4] Lindgren I., Lexell J., Jonsson A.-C., Brogardh C. (2012). Left-sided hemiparesis, pain frequency, and decreased passive shoulder range of abduction are predictors of long-lasting poststroke shoulder pain. *PM & R : The Journal of Injury, Function, and Rehabilitation*.

[5] Zeilig G., Rivel M., Doron D., Defrin R. (2016). Does hemiplegic shoulder pain share clinical and sensory characteristics with central neuropathic pain? A comparative study. *European Journal of Physical and Rehabilitation Medicine*.

[6] Chantraine A., Baribeault A., Uebelhart D., Gremion G. (1999). Shoulder pain and dysfunction in hemiplegia: effects of functional electrical stimulation. *Archives of Physical Medicine and Rehabilitation*.

[7] Faria-Fortini I., Michaelsen S. M., Cassiano J. G., Teixeira-Salmela L. F. (2011). Upper extremity function in stroke subjects: relationships between the international classification of functioning, disability, and health domains. *Journal of Hand Therapy*.

[8] Lindgren I., Jonsson A.-C., Norrving B., Lindgren A. (2007). Shoulder pain after stroke: a prospective population-based study. *Stroke*.

[9] Wanklyn P., Forster A., Young J. (1996). Hemiplegic shoulder pain (HSP): natural history and investigation of associated features. *Disability and Rehabilitation*.

[10] Jadad A. R., Moore R. A., Carroll D. (1996). Assessing the quality of reports of randomized clinical trials: is blinding necessary?. *Controlled Clinical Trials*.

[11] Gronsbell J., Hong C., Nie L., Lu Y., Tian L. (2020). Exact inference for the random-effect model for meta-analyses with rare events. *Statistics in Medicine*.

[12] Whitehair V. C., Chae J., Hisel T., Wilson R. D. (2019). The effect of electrical stimulation on impairment of the painful post-stroke shoulder. *Topics in Stroke Rehabilitation*.

[13] Jang Y. Y., Kim T. H., Lee B. H. (2016). Effects of brain-computer interface-controlled functional electrical stimulation training on shoulder subluxation for patients with stroke: a randomized controlled trial. *Occupational Therapy International*.

[14] Chatterjee S., Hayner K. A., Arumugam N. (2016). The California tri-pull taping method in the treatment of shoulder subluxation after stroke: a randomized clinical trial. *North American Journal of Medical Sciences*.

[15] Mendigutia-Gomez A., Martin-Hernandez C., Salom-Moreno J., Fernandez-de-Las-Penas C. (2016). Effect of dry needling on spasticity, shoulder range of motion, and pressure pain sensitivity in patients with stroke: a crossover study. *Journal of Manipulative and Physiological Therapeutics*.

[16] Backes W. H., Mess W. H., van Kranen-Mastenbroek V., Reulen J. P. H. (2000). Somatosensory cortex responses to median nerve stimulation: fMRI effects of current amplitude and selective attention. *Clinical Neurophysiology*.

[17] del Gratta C., Della Penna S., Tartaro A. (2000). Topographic organization of the human primary and secondary somatosensory areas. *NeuroReport*.

[18] Spiegel J., Tintera J., Gawehn J., Stoeter P., Treede R. D. (1999). Functional MRI of human primary somatosensory and motor cortex during median nerve stimulation. *Clinical Neurophysiology*.

[19] Kampe K. K. W., Jones R. A., Auer D. P. (2000). Frequency dependence of the functional MRI response after electrical median nerve stimulation. *Human Brain Mapping*.

[20] Simons D. J., Land P. W. (1987). Early experience of tactile stimulation influences organization of somatic sensory cortex. *Nature*.

[21] Van der Loos H., Woolsey T. A. (1973). Somatosensory cortex: structural alterations following early injury to sense organs. *Science (New York, N.Y.)*.

[22] Fraser C., Power M., Hamdy S. (2002). Driving plasticity in human adult motor cortex is associated with improved motor function after brain injury. *Neuron*.

[23] Ward N. S., Brown M. M., Thompson A. J., Frackowiak R. S. J. (2006). Longitudinal changes in cerebral response to proprioceptive input in individual patients after stroke: an FMRI study. *Neurorehabilitation and Neural Repair*.

[24] Pasluosta C., Kiele P., Stieglitz T. (2018). Paradigms for restoration of somatosensory feedback via stimulation of the peripheral nervous system. *Clinical Neurophysiology*.

[25] Conforto A. B., Kaelin-Lang A., Cohen L. G. (2002). Increase in hand muscle strength of stroke patients after somatosensory stimulation. *Annals of Neurology*.

[26] Klaiput A., Kitisomprayoonkul W. (2009). Increased pinch strength in acute and subacute stroke patients after simultaneous median and ulnar sensory stimulation. *Neurorehabilitation and Neural Repair*.

[27] Celnik P., Hummel F., Harris-Love M., Wolk R., Cohen L. G. (2007). Somatosensory stimulation enhances the effects of training functional hand tasks in patients with chronic stroke. *Archives of Physical Medicine and Rehabilitation*.

[28] Wu C. W., Seo H.-J., Cohen L. G. (2006). Influence of electric somatosensory stimulation on paretic-hand function in chronic stroke. *Archives of Physical Medicine and Rehabilitation*.

[29] Hayner K. A. (2012). Effectiveness of the California tri-pull taping method for shoulder subluxation poststroke: a single-subject ABA design. *American Journal of Occupational Therapy*.

[30] Yang L., Yang J., He C. (2018). The effect of kinesiology taping on the hemiplegic shoulder pain: a randomized controlled trial. *Journal of Healthcare Engineering*.

[31] Bolognini N., Russo C., Edwards D. J. (2016). The sensory side of post-stroke motor rehabilitation. *Restorative Neurology and Neuroscience*.

[32] Mendigutia-Gomez A., Quintana-Garcia M. T., Martin-Sevilla M. (2020). Post-needling soreness and trigger point dry needling for hemiplegic shoulder pain following stroke. *Acupuncture in Medicine*.

[33] Chau J. P. C., Lo S. H. S., Yu X. (2018). Effects of acupuncture on the recovery outcomes of stroke survivors with shoulder pain: a systematic review. *Frontiers in Neurology*.

[34] Cagnie B., Dewitte V., Barbe T., Timmermans F., Delrue N., Meeus M. (2013). Physiologic effects of dry needling. *Current Pain and Headache Reports*.

[35] Dommerholt J. (2011). Dry needling - peripheral and central considerations. *The Journal of Manual & Manipulative Therapy*.

